# Google trend analysis and lockdown impact on mental health during the COVID-19 pandemic

**DOI:** 10.1192/j.eurpsy.2023.980

**Published:** 2023-07-19

**Authors:** T. Mastelić, T. Borovina Marasović, S. Kozina, T. Glavina

**Affiliations:** Clinic for Psychiatry, Clinical Hospital Centre Split; 2 Psychological medicine, School of Medicine, Split, Croatia

## Abstract

**Introduction:**

The COVID-19 pandemic has affected the mental health of the world’s population in numerous ways. One of the methods are certainly lockdown measures. The countries of the European Union (EU) were affected by the pandemic in different ways, but their response was partly coordinated. Google trend analysis has so far proven to be a useful tool for monitoring the reactions of the population and the state of their mental health.

**Objectives:**

The aim of our research was to examine the impact of lockdown measures on the state of mental health through an internet search for terms related to mental health.

**Methods:**

We observed three countries of the European Union in the period from February 1, 2020. until May 17, 2021. According to the average value of the CSI (covid stringency index), as an indicator of the strength of the lockdown measures, we chose Estonia, Belgium and Italy. CSI uses nine indicators (such as school closures, travel bans, …) to assess the strength of the lockdown. Italy is the country that, according to the average value of the CSI, had the strongest closure measures in the mentioned period (average CSI 69.19468). In Estonia, the measures were the mildest (CSI 42.87324895), and Belgium represents the average (CSI 57.6381). We observed to what extent, in the mentioned countries, changing the CSI, i.e. the strength of lockdown measures, correlates with the search for terms in the field of mental health. We used Google trends data for the terms: tension, anxiety, depression, insomnia, concern. We also compared Croatia with the mentioned countries.

**Results:**

In Estonia, there is no significant correlation between lockdown measures and searches for mental health terms. In Belgium, there is a correlation between CSI and searches for the term “anxiety” (r=0.31, p<0.01). In Italy, there is a correlation between CSI and searches for the terms “concern” (r=0.22, p=0.067 ), “tension” (r=0.33, p<0.01), “anxiety” (r=0.55, p<0.001). In Croatia, which is the 4th country with the weakest lockdown measures (CSI 46.90232), there is only a correlation between the strength of lockdown measures and searches for the term “tension” (r=0.27, p<0.05).

**Conclusions:**

In countries with a higher CSI, i.e., stronger lockdown measures, there is a stronger correlation, i.e., a greater influence on the search for terms related to the state of mental health. We can assume that at some point the lockdown measures start to have a negative effect on the state of mental health.

**Disclosure of Interest:**

None Declared

